# Congenital Lung Malformations: Unresolved Issues and Unanswered Questions

**DOI:** 10.3389/fped.2019.00239

**Published:** 2019-06-13

**Authors:** Federica Annunziata, Andrew Bush, Francesco Borgia, Francesco Raimondi, Silvia Montella, Marco Poeta, Melissa Borrelli, Francesca Santamaria

**Affiliations:** ^1^Division of Paediatrics, Department of Translational Medical Sciences, Federico II University, Naples, Italy; ^2^Department of Paediatrics and Paediatric Respiratory Medicine, Imperial College and Royal Brompton Hospital, London, United Kingdom; ^3^Divisions of Cardiology and Cardiothoracic Surgery, Department of Advanced Biomedical Sciences, Federico II University, Naples, Italy

**Keywords:** lung malformations, pulmonary sequestration, congenital cystic adenomatoid malformation, bronchogenic cyst, postnatal management, surgery, children

## Abstract

Advances in prenatal and postnatal diagnosis, perioperative management, and postoperative care have dramatically increased the number of scientific reports on congenital thoracic malformations (CTM). Nearly all CTM are detected prior to birth, generally by antenatal ultrasound. After delivery, most infants do well and remain asymptomatic for a long time. However, complications may occur beyond infancy, including in adolescence and adulthood. Prenatal diagnosis is sometimes missed and detection may occur later, either by chance or because of unexplained recurrent or persistent respiratory symptoms or signs, with difficult implications for family counseling and substantial delay in surgical planning. Although landmark studies have been published, postnatal management of asymptomatic children is still controversial and needs a resolution. Our aim is to provide a focused overview on a number of unresolved issues arising from the lack of an evidence-based consensus on the management of patients with CTM. We summarized findings from current literature, with a particular emphasis on the vigorous controversies on the type and timing of diagnostic procedures, treatments and the still obscure relationship between CTM and malignancies, a matter of great concern for both families and physicians. We also present an algorithm for the assessment and follow-up of CTM detected either in the antenatal or postnatal period. A standardized approach across Europe, based on a multidisciplinary team, is urgently needed for achieving an evidence-based management protocol for CTM.

## Introduction

The term congenital lung malformation is used as an umbrella term to cover a wide range of disorders, that include the entity formerly known as congenital cystic adenomatoid malformation (CCAM), intra- and extra-lobar pulmonary sequestration (PS), bronchogenic cysts, congenital large hyperlucent lobe (CLHL, also reported as congenital alveolar overdistension, formerly known as congenital lobar emphysema) and bronchial atresia. In the international literature the term “congenital thoracic malformations” (CTM) has been introduced as a term to describe the above entities clinically (1, 2). Clearly more sophisticated classifications should be used by pathologists examining excised CTM. We will use this terminology throughout the manuscript.

CTM account for 5–18% of all congenital abnormalities, have a cumulative incidence of 30–42 cases per 100,000 individuals (3), and thus are considered rare disorders (4). However, the prevalence may be underestimated as an unknown proportion of these lesions is detected postnatally by chance (5).

Complications of CTM were first reported in the 1970s (6). In the last two decades, antenatal diagnosis has been the rule, and improved postnatal imaging has detected missed cases (5). CTM are considered in the differential diagnosis of recurrent pneumonia occurring in the same location in children (7).

Despite multiple publications on the topic, there are actually no uniform management guidelines, and the postnatal management of CTM differs markedly among centers (8–16). This deficiency greatly hampers parental counseling, including pregnancy decisions and surgical planning.

Herein we review the existing literature on CTM. Our main objectives were (i) to summarize their etiology and classification, (ii) to describe their clinical presentation and associated complications, (iii) to review the evidence on the diagnostic approach and therapeutic strategies, and (iv) to highlight unanswered management questions. We carried out a literature search for English articles published on this topic since 1990 up to May 2019, in the Scopus, Web of Science, PubMed, and MEDLINE databases, using the search term “congenital lung malformations,” “congenital thoracic malformations,” “pulmonary sequestration,” “congenital cystic adenomatoid malformation,” “congenital pulmonary adenomatoid malformation,” “congenital large hyperlucent lobe,” “congenital alveolar overdistension,” “congenital lobar emphysema,” “bronchogenic cyst,” combined with the following: “postnatal management,” “surgery,” “embolization.” We excluded studies conducted exclusively in adults, but included those with a mixed study population of children (or adolescents) and adults. We have developed an algorithm for the evaluation and follow-up of cases of CTM detected either in the antenatal or postnatal period.

## Etiology and Classifications of CTM

Several hypotheses on the etiology of CTM have been proposed. It has been speculated that they are due to anomalies of airway embryogenesis, with both type and histopathology being related to the timing of the embryologic insult (17–22). This unifying theory might explain the overlapping features often seen in CTM; however, embryological speculations are often incorrect! An animal study suggested also that an exaggerated signaling of the fibroblast growth factor 10 (FGF10) may be responsible for the formation of abnormal cystic-like structures during early lung development (17). FGF10 contributes to lung morphogenesis through its receptor FGFR2, and its signaling is down-regulated by the Sonic Hedgehog (SHH) system. The deregulation of SHH-FGF10 signaling has been hypothesized to be the cause of CCAM (17). Moreover, a recent study showed that CTM consist of differentiated airway structures transcriptionally characterized by increased expression of airway epithelial markers as well as by dysregulated expression of genes related to the Ras and several kinases signaling pathways (23). A murine study showed that mutations in the *DICER1* gene leads to the formation of cystic airways, disruption of branching morphogenesis and mesenchymal expansion, features similar to pleuropulmonary blastoma (PPB) (24).

There is currently debate about the pathological classification of CTM. According to the 2002 Stocker classification, the term congenital pulmonary airway malformation (CPAM), that replaced the former CCAM, includes five types (1). Type 0 CPAM (bronchial type, formerly described as acinar dysplasia) is characterized by bronchial-type airways separated only by abundant mesenchymal tissue. Types 1 (the bronchial/bronchiolar type) and 2 CPAM (the bronchiolar type) are characterized by cysts >2 cm in diameter and multiple small cysts, respectively. In type 3 CPAM (the bronchiolar/alveolar type) the lesion is solid, and not cystic, because of the excess of bronchiolar structure separated by airspaces that resemble late fetal lung, while type 4 CPAM (the peripheral type) is characterized by peripheral thin-walled, often multiloculated cysts (2, 5, 8). However, the utility of classifications is different depending on whether obstetricians, pediatricians, radiologists, pathologists, or surgeons assess a CTM (1, 25–27). Although pathological classifications can be established only through histological examination, there is a great need to develop a uniform phenotypic description, particularly because pathological features of more than one lesion may be present in the same case and many diagnoses based on imaging have to be revised after pathological evaluation (2), hence the logic of the use of the umbrella term CTM.

Up to 6% of all CTM are PS (3), a malformation with no communication with the bronchial tree (8). Arterial blood supply is usually from the thoracic or abdominal aorta, or occasionally from other arteries (8). Extralobar PS, which has its own pleural lining, is usually situated below the left lower lobe (subdiaphragmatic site, 15% of the cases), and is less common than intralobar PS, which is mainly located within the left lower lobe. Mixed PS and CPAM is defined as a hybrid lesion, and is common in cases with extralobar PS (8, 28–30). Bronchogenic cysts, the most common isolated cyst reported in infancy, are situated in ~50% of the cases in the mediastinum, close to the carina, and are characterized by closed respiratory-type epithelium-lined sacs containing cartilage in the wall developing from the primitive respiratory tract (2). CLHL most commonly affects the left upper or right middle lobes, and may show very few primitive alveoli or even a polyalveolar lobe. Mechanisms proposed to explain the air-trapping include dysplastic or deficient bronchial cartilage, thick mucus, extensive mucosal proliferation, bronchial torsion, bronchial atresia, and bronchial compression by cardiopulmonary vessels, lymph nodes, cysts, polyalveolar lung, or focal pulmonary hypoplasia (30). Overall, bronchogenic cysts, CLHL and other malformations (bronchial atresia, congenital small lung, and absent lung or trachea) have a significantly lower incidence than CPAM and PS (from 1:20.000 to <1 per 100.000 live births) (31–36).

Obviously, pathological classification of CTM is available only after surgery or at autopsy (1, 26, 27). Clinicians have to rely on gray scale images, namely prenatal ultrasound (US) and possibly magnetic resonance imaging (MRI), the first postnatal lung imaging findings and any associated clinical features. Hence a clinical and imaging classification has been proposed which has the advantage of being derived from widely available investigations (25).

## Clinical Presentation and Complications Associated with CTM

Most CTM are detected prior to birth at prenatal US (37). The cystic and/or solid lesion may progressively enlarge, with eventual mediastinal shift, or also regress totally or partially before birth (38, 39). Antenatal complications of CTM include fetal hydrops, pleural effusion, or polyhydramnios secondary to failure of normal fetal swallowing because of esophageal compression. Hydrops, reported in 5–30% of all CTM, is the gravest complication, associated with high mortality (40, 41), and therefore requires prompt prenatal intervention and/or preterm delivery (10).

At birth, clinical presentation of CTM is variable. Delivery is usually uncomplicated. Most neonates (>75%) are asymptomatic, with only a minority requiring any respiratory support (12). Beyond the neonatal period, presentation relates to infections and chronic cough or recurrent wheeze, although most babies remain asymptomatic. Symptoms are reported at an average age of 7 months (10). However, many are non-specific childhood complaints and unlikely related to the CTM. In some cases, a CTM might be suspected because of the coexistence of extrapulmonary anomalies, especially in patients with PS who may have associated congenital diaphragmatic hernia or an additional CTM, as well as cardiovascular abnormalities (25, 42).

Potential postnatal complications of undetected or untreated CTM include infections (bacterial, and also fungal and mycobacterial), bleeding (which may lead to hemothorax), air embolism, high-output cardiac failure due to shunting through systemic collaterals, pneumothorax and malignant changes (7, 8, 25, 27). It has been reported that these complications occur in about 3.2% of non-operated patients (43, 44). Although some complications may be prevented by prophylactic surgery, even complete resection of the lesion cannot preclude malignancy arising in the remaining lung tissue (13, 39, 45, 46).

The relationship between CTM and the development of malignancies is debated. PPB, bronchioloalveolar carcinoma and lung adenocarcinoma have been associated with CTM (44, 46). PPB is rare in the general population, with an incidence of 1 in 250,000 live births, but the frequency rises up to 4% in children with CPAM, and its mortality rate is about 20% (47–49). It is unclear whether type 4 CPAM is a regressed PPB or rather PPB is a complication of type 4 CPAM. Cavitation may be secondary to tumor necrosis or, conversely, malignancies may develop within cysts. Indeed, PPB has been described as a distinct entity, with similarities in imaging if compared to CPAM, but with its own specific genetic and molecular markers (24). Factors favoring the diagnosis of PPB include some specific features such as the development of pneumothorax, the evidence of bilateral or multisegment involvement and of a complex cyst, and, finally, a germline mutation in the *DICER1* gene, whereas CPAM is more likely with prenatal diagnosis, and the presence of a systemic feeding vessel and hyperinflated lung (24). A recent study on gene expression in CPAM also including mucin-encoding genes, found that *K-RAS* mutations and *MUC5AC, CK20*, and *HER2* expression genes (involved in early lung adenocarcinoma development) were present in all CTM with mucinogenic proliferation, thus supporting the importance of complete surgical resection of CTM because of the possible neoplastic nature of at least type 1 CPAM (50). These data provide further insights into the hypothesis that intra-cystic mucinous proliferation, typically seen in type 1 CPAM, may be the precursor also of the bronchioloalveolar carcinoma (49, 51). There are also reports of PPB and bronchioloalveolar carcinoma in children and adults with resected bronchogenic cysts (14, 52), and, finally, a preexisting bronchogenic cyst has been frequently associated with the development of pulmonary adenocarcinoma in adulthood (53). In conclusion, there is still a lively debate on the relationship between malignancies and CTM, indicating that the issue needs to be further investigated before a definite conclusion is reached.

## Evidence on the Diagnostic Approach and Therapeutic Strategies for CTM

### Diagnostic Approach

Antenatally, close monitoring with serial fetal US is the only investigation usually performed to assess size, location, characteristics (i.e., macro- or micro-cystic, solid, or mixed lesions), and volume changes with growth, as well as blood supply (although small accessory vessels arising below the diaphragm can be missed), mediastinal shift, pleural effusion, or other signs of fetal hydrops. Currently, it is impossible to predict accurately the behavior of a CTM *in utero*. In many cases, the lesion may be invisible at term (25). Although regression of the lesion after the 30th week of pregnancy is common, postnatal assessment is always recommended (8). Antenatally, if the lesion progressively enlarges and/or there are no signs of regression in the last 10 weeks of pregnancy, or if hydrops develops, options for intervention should be considered (25). In addition to US, fetal MRI may also be useful both to detect a systemic arterial blood supply and any complications. Moreover, a lesion volume >24.0 cm^3^ at MRI during the third trimester of pregnancy has a 100% sensitivity and 91% specificity in predicting neonatal respiratory distress (54).

For cystic lesions, the CTM volume ratio (CVR) is a useful prognostic tool (55). It measures the volume of the lung lesion, divided by the head circumference to normalize for gestational age. A CVR >1.6 predicts an 80% increased risk of fetal hydrops, while a ratio <1.6 is associated with a survival rate of 94% and risk of hydrops <3% (56). Recently, it was suggested that a CVR ≥0.84 is a good predictor of respiratory distress at birth, as well as other US findings including polyhydramnios and ascites (57). The mass-to-thorax ratio is an additional good predictor of adverse events, with also a negative predictive value of the risk of developing hydrops if >0.96 (58, 59). Finally, the increase of the cardiomediastinal shift angle, a novel measure of mediastinal shift, has been significantly associated with an adverse perinatal outcome of CTM (60).

Prenatal imaging is not a reliable predictor of post-natal histology. For example, the prenatal demonstration of a systemic arterial supply to a CTM, although generally associated with PS, is also found in hybrid lesions (33). Moreover, failure to detect a systemic arterial supply does not exclude PS (5).

**Figure 1:**
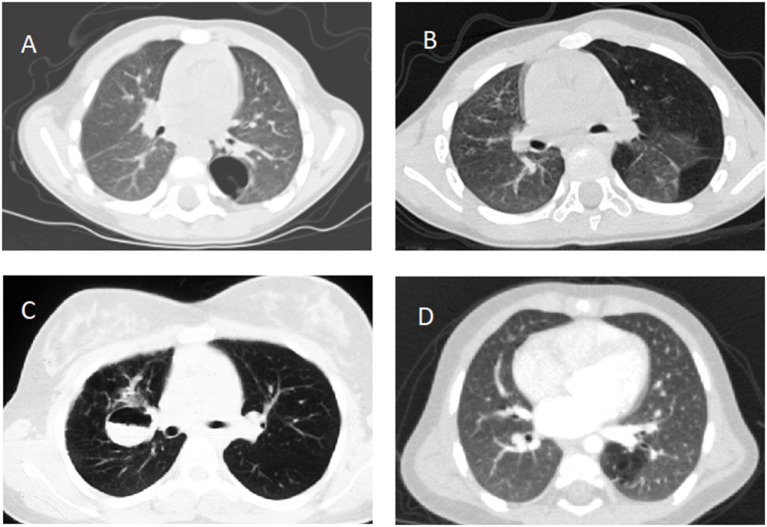
Computed tomography scans documenting **(A)** type 1 congenital pulmonary airway malformation in the left lower lobe, **(B)** congenital lobar emphysema involving the left lung, **(C)** fluid filled bronchogenic cyst in the right lung, **(D)** intralobar pulmonary sequestration in the left lower lobe, all confirmed after lobectomy except **(B)**.

Although CTM are usually detected antenatally, the diagnosis may be missed until later in life. In those detected antenatally, a chest radiograph is performed in many centers shortly after birth, and this is often normal. However, chest radiography has low sensitivity for detecting CTM (11). Therefore, initial investigation should include a computed tomography (CT) scan within the first months of life to confirm that the suspected CTM is still present, and many would propose the use of contrast to delineate the arterial supply and venous drainage prior to surgery, especially if PS is suspected (10, 11, 13, 14) (Figure 1). However, the timing of the first HRCT is still controversial. MRI may be a good alternative to CT to avoid radiation exposure, though MRI currently has a long image acquisition time, and thus requires the patient to be cooperative or sedated/anesthetized (61). Furthermore, MRI may not detect thin-wall cysts and emphysematous changes as well as CT (8, 62). Finally, MRI is not universally available and requires expertise in interpretation. However, MRI may be better than CT in mapping vascular anatomy, especially if the vessel is small and the drainage is close to the cardiac cavity (8).

### Therapeutic Strategies for CTM

Although most CTM have a favorable prognosis, with a survival rate >95% (40, 41, 63), there is a risk of antenatal and postnatal complications (7, 45). When antenatal complications occur, the possible therapeutic options include thoracocentesis, pleuro-amniotic shunt placement, percutaneous ultrasound-guided sclerotherapy, or radiofrequency/laser ablation, fetal bronchoscopy, and rarely open fetal surgery (8, 10, 25, 64, 65), none of which are evidence based, and all of which should be considered a last resort. Factors that should be taken into account are gestational age, the position of the fetus and placenta, whether macrocysts or a high-flow vascular component is present, and, most of all, whether there has been a referral to a center with qualified multidisciplinary team and expertise. When fetal lung maturity is felt sufficient to provide good chances of postnatal survival, preterm delivery may represent a reasonable choice (66). If prior to postnatal viability fetal demise appears likely, repeated cyst aspiration and thoraco-amniotic shunting are therapeutic options. It has been reported that fetal bronchoscopy may represent a therapeutic tool with good outcome, but only in specialized centers (65, 66). Maternal betamethasone administration during the second trimester of gestation has been demonstrated to induce regression of some CTM and reverse fetal hydrops, increasing survival rate (67, 68). Steroids decrease the production of lung fluid and increase its reabsorption within the CTM, thus mimicking the physiological third trimester changes (69). They are indicated for microcystic lesions, while it is unclear whether macrocytic CTM respond to this treatment (70, 71).

If symptoms and/or complications develop postnatally, the child should first be stabilized as far as possible and then therapeutic decisions taken. With the technological improvements in minimally invasive surgery, CTM are now usually removed by video-assisted thoracoscopy (VATS), a safe and feasible alternative to open thoracotomy (15, 72). VATS is usually uncomplicated, and allows the compressed surrounding lung to expand. Advantages of VATS over thoracotomy include smaller incisions with obvious cosmetic benefits, less pain, slightly lower complication rates, shorter hospital stays albeit with longer operative time, and more rapid return to normal activity (73–75). Moreover, the magnification provided by VATS allows for significantly improved discrimination between normal and affected lung and better visualization of fissures and vascular structures. Lobectomy is recommended for the majority of parenchymal CTM referred for surgery, in order to prevent postoperative air leaks, residual disease, and perhaps reduce the risk of some later malignancies (14). Conversely, lung-sparing strategies such as segmentectomy have been advocated for small, well-defined segmental lesions and in cases with bilateral or multilobar disease (75–77). Malignancy even after apparent complete resection of a CTM has been described in the same or also different lung areas (2, 78), but it cannot be excluded that the CTM was indeed neoplastic from the beginning (24).

**Figure 2:**
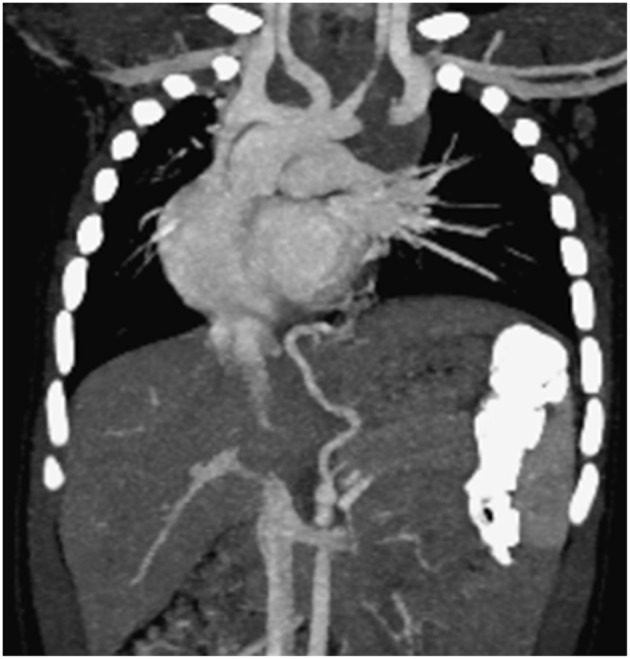
Computed tomography scan documenting two aberrant vessels originating from the tripod celiac artery that lead to the left lower lobe representing intralobar pulmonary sequestration (coronal view).

**Figure 3:**
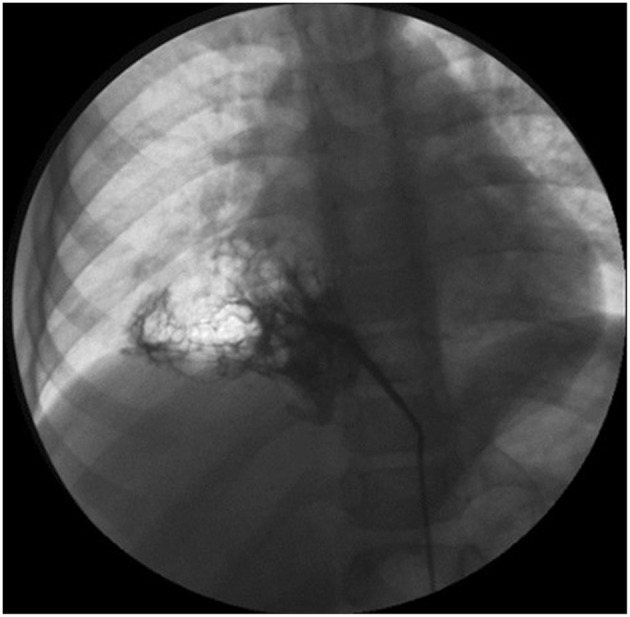
Angiography showing an aberrant artery originating from the celiac trunk to the right lower lobe.

If a systemic arterial blood supply is demonstrated by CT (Figure 2) or MRI, or PS is suspected, and embolization of the feeding vessel is contemplated, angiography should be performed to confirm the presence of abnormal vessels, assess their size and course, and guide transcatheter embolization (79) (Figure 3). Embolization leads to regression or complete involution of at least the solid components of CTM, as well as correcting high output cardiac failure if this is present (75), and thus is the preferred therapeutic option in this setting. Several embolization techniques have been proposed, and vascular plugs or microcoils are preferred to injection of alcohol, histoacryl, or gelatin sponge particles (80). Complications after percutaneous embolization are very rare, including migration of the occlusive device, infection, pain, and fever. Although embolization is an acceptable therapeutic strategy, there is still no clear consensus on which CTM are a good indication for first-line embolization. Moreover, cases referred for embolization should be carefully selected as secondary surgery was recently shown to be necessary in 13% of embolized children (81). Hybrid CTM with feeding vessels and other CTM and duplication cysts are at risk of infection or cancer, and should preferably undergo surgical resection (82). If there are large or multiple arteries, re-embolization may be necessary (81). Nevertheless, for carefully selected cases, embolization is a possible option, but long-term data are necessary to confirm clearly the indications for this procedure.

If imaging suggests a CLHL, postnatal management is conservative, unless there are symptoms. This lesion usually regresses over time, and there is no evidence that the development of the underlying lung is improved by surgery. However, if the enlarged lobe causes respiratory symptoms in the newborn period, lobectomy is indicated (8, 14).

While there is general consensus that any symptomatic CTM should be treated, the best management of asymptomatic children is controversial. The divergent opinions on type and timing of any procedure for CTM reported in the literature are summarized in the Table 1. Treatment may be conservative or surgical. If an initial “wait and see” approach is adopted (13, 87), lung imaging may be repeated at 18 months of age, and if the CTM is confirmed, surgery should be planned around the 2nd birthday to allow the baby to grow (13). The rationale for this is to allow for possible spontaneous regression of the CTM, and thus a conservative approach is proposed until symptoms occur or when the cyst size changes on lung imaging (15, 16, 88, 89). Some authors follow-up asymptomatic patients by yearly physical examination and CT scans (16), but the radiation burden of this approach is not trivial. Moreover, the conservative approach has several issues including the risk of complications developing, of losing patients to follow-up while waiting, of increased surgical morbidity if the CTM becomes infected, or also of a substantial radiation exposure due to repeated CT (15, 91). However, the proportion of cases in whom, within the first year of life, the CTM become infected or regress spontaneously is variable (15, 16, 43, 92). The mean follow-up of patients treated conservatively is insufficient to confirm that this is the best option, as malignancies may occur much later, even in adulthood (16).

**Table 1:** Summary from the literature on the diagnostic work up and therapeutic strategies to antenatal suspicion of CPAM and PS in asymptomatic infants.

**Diagnostic work up**	**Therapeutic strategies**			
**Procedure and timing**	**Operative approach and timing**		**Conservative approach**	
	**CPAM**			
	Elective surgery in all cases, within the 1st year of life (11, 12, 22, 83–86) Or In cases with large and medium-sized cysts, within the 1st year of life (14, 87) Or		Only in cases with small-sized cysts (87, 88) Or Until symptoms occur or changes in size are observed radiologically or parents/patients have concern (at which time surgery is due) (15, 16, 89)	
**Chest X-ray, shortly after birth (11, 12, 14, 24)**	In all cases up to 18 months of age when a 2nd HRCT is made for confirming the lesion (13)	
	**PS**			
**HRCT, within the first months (11, 12, 14, 24)MRI, few weeks after birth (11)2nd HRCT with contrast, at age 12–18 months (24) or at 5 years (89)**	Elective surgery, within the 1st year of life (11) Or after the 1st year of life (81, 90) Or Only for intralobar PS, within the 1st year of life (14) Or In all cases up to 18 months of age when a 2nd HRCT is made for confirming the lesion (13) Or Elective embolization in case of CTM with no symptoms and no cysts (81)		Extralobar PS without significant shunting (14) Or Until symptoms occur or changes in size are observed radiologically or parents/patients have concern (at which time surgery is due) (89)	
	**Advantages**	**Disadvantages**	**Advantages**	**Disadvantages**
**3rd HRCT, prior to transition (89)**	**Less risk of late complications Less risk of emergency surgery Prevention of cancer in the lesion itself More time for lung growth Short/long-term normal lung function**	**Potential operative morbidity and mortality No prevention of cancer in other areas of the lung**	**Avoidance of surgery if the lesion regresses spontaneously**	**Risk of complications during “wait and see” period Risk of developing high-flow heart failure Pulmonary Hypertension Abnormal lung growth Cumulative radiation risk Risk of losing patients to follow-up Greater morbidity of emergency surgery**

*CPAM, Congenital Pulmonary Adenomatoid Malformation; PS, Pulmonary Sequestration; HRCT, High Resolution Computed Tomography*.

Other authors advocate elective surgery as the preferred option for a number of reasons, including prevention of late infection or cancer, less risk of emergency surgery, and more time for compensatory alveolar growth (11, 12, 22, 83, 84, 90). However, there are potential operative risks, albeit low in centers with expertise and experience, and cancer may develop elsewhere in the lungs despite the resection of the CTM (45). Also, there is no consensus on the age at surgery, with some preferring to operate in the neonatal period (93), and others waiting until after 4 weeks of age to reduce the risks of the anesthesia prior to that age (22). A recent retrospective study aimed to determine the optimal timing for CTM resection within the first year of life did not find significant differences in the complication rates, hospital re-admissions, or conversion from VATS to open surgery, suggesting that surgery is equally safe whenever made from the first month of life until the first birthday (85). Other authors concluded that morbidity associated with surgery is significantly higher in infants younger than 3 months, and suggested that the optimal timing is 3–9 months of life, as the surgical intervention duration significantly increases in older infants (86).

## Unanswered Questions about the Management of CTM

CTM represent a heterogeneous group of abnormalities. Although the number of cases suspected or diagnosed early has been increasing (94), many management questions remain unanswered, and there are no universally accepted clinical recommendations or practice guidelines.

In terms of diagnosis, although prenatal US and postnatal CT scan are currently considered the gold standard tests, MRI is increasingly used for diagnosing CTM both antenatally and postnatally (5, 40, 95). Postnatal MRI has also been used as radiation-free technique to study any vascularization or re-vascularization after embolization of a systemic arterial supply (79). Nevertheless, the long scanning time and the scanner noise during the examination usually means that general anesthesia is required in infants older than 6 months of age, when the “feed and wrap” technique may be precluded. Future technological improvements will likely overcome these limitations. Widening the indications of chest MRI to suspected CTM would hopefully clarify whether or not MRI is a reliable tool for their diagnosis postnatally, thus reducing the extra radiation exposure associated with CT (15).

**Figure 4:**
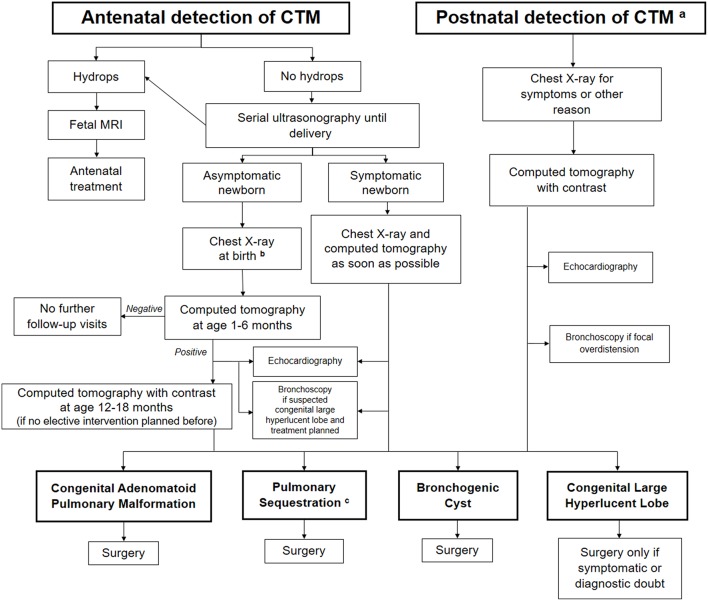
Proposed algorithm for the assessment and follow-up of congenital thoracic malformations (CTM) detected either in the antenatal or postnatal period. ^a^Postnatal detection may be suggestive of pleuropulmonary blastoma as well. ^b^A Computed Tomography (CT) should be performed to detect a possible lesion at age 1–6 months in all asymptomatic cases, whatever is the chest X-ray finding (positive or negative) at birth. ^c^Embolization considered only in asymptomatic cases without evidence of cysts.

Whether conservative or active treatment of asymptomatic patients is best is still controversial, mainly because of the lack of knowledge on the natural history of CTM (8–16, 93). There is no reliable evidence on the optimal management of affected children. Indeed, most babies diagnosed with a CTM do well in the medium term, but an undefined proportion may develop malignancy. At present, this high-risk group cannot be identified, and therefore prospective studies gathering data to try to address this situation are urgently needed. A proposal to detect this high-risk group that includes radiological features and *DICER1* mutation analysis has been made (2). The indications for surgery and the timing in asymptomatic children with CTM is also controversial, highlighting the need for long-term outcome studies involving large numbers of patients. The risk of repeated radiological studies and the problem of losing patients at follow-up during a “wait and see” period must also be taken into account. Due to the divergent opinions on the management of CTM and its relevant impact on the family expectations and healthcare costs, we propose a diagnostic-therapeutic algorithm (Figure 4), which may be helpful for clinicians dealing with detection of CTM either in the antenatal or postnatal period. Like all algorithms, it is not meant to replace clinical judgment, but it should rather drive physicians to adopt a systematic approach to CTM.

Large-scale, prospective databases with data on clinical presentation, treatment and long-term course of CTM would be a good option to develop a shared clinical guideline. Although expert opinion should be kept in mind (8–16), the time has come to find answers to the unresolved issues of CTM by gathering evidence (96, 97). As controversies sill arise in this field, and many questions about proper management and follow-up are unanswered, a global CTM registry should be designed which would hopefully represent a new promising tool to advance the understanding of these rare disorders, to recruit candidates for research studies and ultimately to improve care of patients with an asymptomatic CTM.

## Author Contributions

FA, MP, and MB drafted the initial manuscript, searched for bibliography, and revised the final manuscript. FR, FB, and SM were involved in drafting the manuscript, critically revised the manuscript, and approved the final manuscript. AB and FS made substantial contributions to conception and design of the study and reviewed and approved the final manuscript. All authors read and approved the final manuscript as submitted.

### Conflict of Interest Statement

The authors declare that the research was conducted in the absence of any commercial or financial relationships that could be construed as a potential conflict of interest.
